# Targeted drug delivery systems for cardiovascular disease treatment: principles, targeting strategies, and future prospects

**DOI:** 10.1080/07853890.2026.2667553

**Published:** 2026-05-11

**Authors:** Xianyun Shao, Junhao Wen, Ziqing Yan, Xingyu Su, Wan Lin, Hang Li, Haiyan Yin, Senquan Wu, Yiyang Wang

**Affiliations:** a Department of Pathophysiology, School of Medicine, Jinan University, Guangzhou, China; b Key Laboratory of State Administration of Traditional Chinese Medicine of the People’s Republic of China, School of Medicine, Jinan University, Guangzhou, China; c Department of Life Science and Medical Bioscience, Laboratory for Molecular Brain Science, Waseda University, Tokyo, Japan; d Intensive Care Unit, The First Affiliated Hospital of Jinan University, Guangzhou, China; e Department of Respiratory and Critical Care Medicine, Dongguan People’s Hospital, Dongguan, China

**Keywords:** Administration routes, cardiovascular disease, targeted delivery carriers, targeted drug delivery systems, targeted therapy, targeting mechanism

## Abstract

**Background:**

Targeted drug delivery systems achieve precise drug enrichment at lesion sites through mechanisms such as passive targeting, active targeting, and stimulus-responsive release, offering novel pathways to enhance therapeutic efficacy while reducing systemic toxicity. With advancements in technologies like antibody-drug conjugates and carrier-based encapsulation, these systems have demonstrated remarkable success in treating multiple diseases.

**Discussion:**

In the field of cardiovascular diseases (CVD), the large patient population and persistently high mortality rates, coupled with limitations of conventional drugs and surgeries, such as narrow therapeutic windows, insufficient targeting precision, and high invasiveness, make the development of novel targeted delivery strategies crucial for overcoming current therapeutic bottlenecks. Proteins and small molecule drugs are regarded as ideal candidates for targeted delivery due to their high specificity, favorable stability, and tissue penetration capabilities, while delivery carriers such as exosomes and liposomes play a central role in protecting drug activity and guiding targeted accumulation. Furthermore, the appropriate selection of administration routes significantly influences the efficacy of targeted delivery and clinical outcomes. At present, there is still a lack of systematic summaries on the application of targeted drug delivery systems composed of proteins and small molecule drugs in cardiovascular diseases.

**Conclusions:**

This review focuses on targeted drug delivery systems for CVD, to systematically examine the functional properties of carriers designed for proteins and small molecule drugs, as well as the efficacy of different administration routes. It also offers a forward-looking perspective on future trends, aiming to provide theoretical and practical insights for precision medicine.

## GRAPHICAL ABSTRACT

**Figure:**
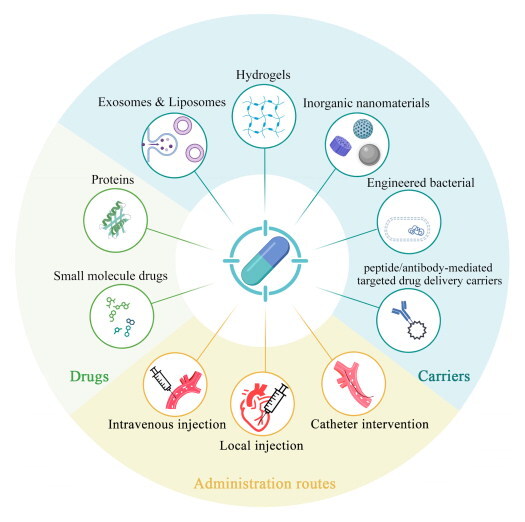

## 1. Introduction

In the process of drug development, improving efficacy and reducing side effects are always the two core goals pursued by researchers. Traditional drugs can enhance the specificity and affinity of the target by optimizing the molecular structure, while reducing the binding to the non-target site, so as to improve the efficacy and reduce the side effects. However, even with precision design, it is difficult for some drugs to avoid affecting normal tissues. For example, steroidal anti-inflammatory drugs, because of their broad physiological effects, produce effects in multiple tissues throughout the body, making it difficult to harmonize the conflict between enhancing efficacy and controlling side effects. The emergence and development of targeted drug delivery systems provide a new direction for solving this conflict. After a long period of technical exploration, targeted therapy was not formally applied in clinical practice until the approval of the first targeted drug rituximab (Rituxan) in 1997, which opened up a new chapter of targeted drug delivery systems [1]. Targeted drug delivery therapy represents a precision medicine approach, the core of which is to concentrate drugs in targeted tissues, thereby improving symptoms while reducing the impact on healthy tissues [2]. This strategy primarily encompasses three targeting mechanisms: passive targeting leveraging pathophysiological characteristics unique to diseased tissues; active targeting relying on surface-modified carrier-bound ligands; and stimulus-responsive targeting utilizing carriers that respond to environmental signals [3–5]. Therefore, in modern medicine that emphasizes precise treatment, targeted drug delivery systems are playing an increasingly important role.

At present, a variety of targeted therapy drugs have been successfully applied in clinical practice, bringing new hope to many patients, among which the development of anti-tumor drugs is particularly rapid. Since the approval of rituximab by the U.S. FDA opened the door to targeted therapy, antibody-drug conjugates (ADCs), as an innovative therapeutic approach, have gradually gained prominence. By integrating the high targeting specificity of antibodies with the potent cytotoxicity of cell-killing agents, ADCs demonstrate remarkable therapeutic potential. The representative drug Trastuzumab Deruxtecan (T-DXd) is guided by anti-HER2 antibody (trastuzumab) to accurately identify HER2-positive breast cancer cells, and carries the topoisomerase I inhibitor Deruxtecan to achieve precise removal of cancer cells and significantly improve the efficiency of targeted drug delivery [6]. In addition, liposomes can also achieve efficient packaging and precise delivery of drugs. Doxil (Liposomal doxorubicin) is an innovative liposome-encapsulated chemotherapeutic drug, which is commonly used to treat a variety of cancers such as HIV-associated Kaposi’s sarcoma, ovarian cancer and so on [7,8]. In neurodegenerative diseases, drugs targeting degenerated neurons have also provided hope for patients in recent years: Lecanemab (Leqembi) and Donanemab treat Alzheimer’s disease by specifically targeting amyloid-beta (Aβ) degradation and eliminating amyloid plaques in neurons [9,10]. These targeted therapeutics that have been successfully applied in clinical practice have not only significantly enhanced therapeutic efficacy and reduced adverse effects but have also successfully transformed numerous previously refractory diseases into manageable conditions. Building on these advances, it is reasonable to believe that targeted drug delivery systems hold broad development prospects for the future. Particularly in the field of cardiovascular diseases (CVD), they offer a promising exploratory space for targeted therapy.

The World Heart Federation (WHF) stated that more than 500 million people worldwide were affected by CVD, and that global cardiovascular disease-related mortality has increased [11]. The current conventional drug treatment options for CVD remain inadequate due to several shortcomings, including narrow therapeutic window, low biological efficacy (first-pass effect), and lack of targeting and drug resistance [12–15]. Concurrently, surgical treatment is also restricted in clinical application due to its high traumatic nature, limited indications, and elevated requirements for postoperative recovery and care [16–18]. In this context, targeted drug delivery system is expected to open up a new path for the treatment of CVDs, such as myocardial infarction, atherosclerosis, cardiac arrhythmia, diabetic cardiomyopathy, and bring breakthrough progress. Furthermore, after years of development, drug carriers are no longer confined to monoclonal antibodies. Exosomes, liposomes, hydrogels, and inorganic nanomaterials are also efficient carriers for targeted drug delivery systems. These delivery carriers can not only effectively protect drugs from the destruction of the environment *in vivo*, but also guide the drugs to specific target tissues or cells relying on their natural recognition capabilities or through surface modifications and other means, thus reducing systemic side effects. Notably, certain drug carriers can also be designed with specific pore sizes, crosslinking densities, and responsive groups, or cleverly combined with cutting-edge technologies such as ultrasound, to achieve precise control of drug release [19]. Despite advances in delivery carriers, the central determinant of efficacy has always been the drug itself. Among the numerous drugs that can be delivered, proteins and small molecule drugs stand out due to their unique advantages, becoming ideal targeted delivery drugs for the treatment of CVD. Protein drugs possess a high degree of specificity and biological activity, enabling them to recognize and bind to specific targets at the pathological sites, thereby exerting therapeutic effects [20,21]. Meanwhile, such drugs do not require complex steps such as transcription and translation, allowing them to take effect more directly and quickly. Small molecule drugs boast prominent advantages in terms of stability and permeability [22]. Their chemical structures are relatively simple, which renders them less prone to degradation influenced by the complex physiological environment *in vivo*, allowing them to maintain a relatively high blood-drug concentration. Furthermore, characterized by their small molecular mass, they can easily penetrate biological barriers such as the cell membrane, rapidly reaching intracellular targets and exerting their pharmacological effects. A suitable drug administration route directly influences the drug’s therapeutic effects and the extent of side effects. Therefore, selecting the corresponding route of drug administration based on the type of CVD, the characteristics of carriers, and the properties of drugs can better achieve targeted drug delivery, enhance therapeutic effects.

This review focuses on targeted drug delivery systems, using CVD treatment as an illustrative example. It systematically discusses the functional properties of carriers based on proteins and small molecule drugs, as well as the efficacy profiles of different administration routes. Furthermore, it identifies critical hurdles in current targeting strategies and proposes future research trajectories, with the goal of informing and inspiring the next generation of precision therapeutics.

## 2. Rationale for targeted drug delivery

### 2.1. Targeting mechanism

In traditional drug therapy, drugs often face problems such as non-specific distribution, low bioavailability, and large side effects after entering the human body. Targeted drug delivery delivers drugs to the lesion site by specific carriers or technologies, aiming to achieve ‘targeted delivery’ of drugs, significantly improve efficacy, reduce systemic toxicity, and optimize pharmacokinetic properties (half-life, bioavailability, etc.) [23–25]. This technology has become the core direction of modern drug research and development, and its mechanism can be divided into three categories: passive targeting, active targeting and stimulation-responsive targeting [5].

#### 2.1.1. Passive targeting

Passive targeting mechanism does not rely on external ligands or molecular recognition but instead leverage the unique physiological characteristics of diseased tissue, especially the enhanced permeability and retention (EPR) effect [26,27], to achieve natural drug accumulation. Specifically, in certain CVDs, increased vascular permeability and impaired lymphatic drainage enable carriers of an appropriate particle size to extravasate from the circulation and be retained within the diseased tissue *via* the EPR effect [27]. For example, vascular endothelial damage at the site of myocardial infarction (MI) leads to increased permeability, causing the extravasation of plasma fluid and proteins into the infarct tissue, which enhances carrier penetration. Moreover, the compression of lymphatic capillaries by tissue edema blocks lymphatic drainage, thereby delaying drug clearance from the lesion area [28]. Therefore, passive targeting is often used as a foundation for other targeting strategies to improve the overall efficiency of drug delivery systems.

#### 2.1.2. Active targeting

Active targeting mechanism involves modifying specific targeting ligands (such as antibodies, peptides, or glycoproteins) on the surface of the carrier, enabling it to bind with high affinity to overexpressed receptors or biomarkers on the surface of diseased cells, thereby achieving precise drug delivery [29]. The core advantage of this mechanism is its molecular recognition ability, which can significantly increase the drug concentration at the target site and promote active cellular uptake [30]. The mannose-modified carrier system is a successful case [31]. Macrophages highly express mannose receptors on their surface, and mannose-modified carriers can target anti-inflammatory drugs to macrophages in atherosclerotic plaques [31]. However, active targeting also has limitations, such as the potential to induce an immunogenic response. This is especially true when antibody-like exogenous ligands are used, as the body may produce an immune response against them, which can affect the stability and delivery efficiency of the carriers [32]. In addition, the design of active targeting strategies must fully consider the heterogeneity of receptor expression to ensure that the drug can be accurately recognized by and act upon the target cells.

#### 2.1.3. Stimulation-responsive targeting

Stimulus-responsive targeting is an intelligent delivery strategy that triggers drug release based on endogenous or exogenous stimuli [33–35]. The core principle is to use the sensitivity of the carrier to environmental changes, such as pH value, temperature, redox state, or changes in external physical conditions such as light, magnetism, and ultrasound, to regulate drug release behavior [33]. For example, pH-sensitive carriers can release drugs by changing the membrane structure in an acidic environment, while heat-sensitive carriers can achieve precise drug release at specific sites through phase transformation induced by local temperature changes [36,37]. The advantage of this mechanism lies in its high spatial and temporal controllability, which can achieve precise drug delivery at the target site while significantly reducing toxic side effects on normal tissues. However, the design complexity of stimulation-responsive targeting is high, and the response threshold needs to be precisely optimized to ensure effective drug release at the target site while avoiding non-specific release due to an excessive response [38]. In addition, the application of external stimulation may be affected by device limitations or tissue depth, further increasing the difficulty of technical implementation.

## 3. The composition of targeted drug complexes

Composition of targeted drug complexes typically involves two aspects: therapeutic drugs, and carriers. Each of them has a suitable combination in order to play its role best. Here, we will discuss from two aspects: drug molecules and carriers, describing their advantages and applicable scopes.

### 3.1. Proteins and small molecule drugs with delivery potential

#### 3.1.1. Proteins

The development of targeted protein drugs for CVD has primarily focused on the treatment of MI. Among the various targeted protein drugs under investigation, vascular endothelial growth factor (VEGF) has received more attention as a potential treatment for MI. As a key vascular growth factor, VEGF promotes myocardial angiogenesis and establishes new blood vessels to improve myocardial perfusion [39,40], thus reducing the damage caused by ischemia. Moreover, in MI models, VEGF acts synergistically with angiopoietin-1 (Ang-1). The combination of these two factors has been shown to exhibit significant pro-angiogenic effects, which collectively contribute to the enhancement of cardiac function [41]. VEGF also inhibits cardiomyocyte apoptosis and protects cardiomyocytes by activating the PI3K/Akt signaling pathway [42]. However, in consideration of the general function of VEGF, traditional delivery approaches could cause a variety of side-effect, such as oedema, allergy, and even carcinogenesis [43]. So, how to limit the action of VEGF to only the diseased region in order to minimize side-effect is the key topic.

Similarly, targeted delivery of insulin-like growth factor-1 (IGF-1) is a viable treatment option for MI. In addition to its role in promoting neovascularization in the infarct zone by increasing the expression of VEGF and other angiogenic factors [44], IGF-1 has specific functions. For one thing, in the context of cardiac repair following a MI, IGF-1 is not only able to mobilize cardiac stem cells to the infarct site, but to activate the PI3K/Akt and MAPK signaling pathways to inhibit cardiomyocyte apoptosis, thereby promoting cardiomyocyte regeneration and repair [45–47]. For another, IGF-1 enhances the contractility of cardiomyocytes, playing a critical role in improving heart function and increasing contractile efficiency following a MI [48]. Besides MI, IGF-1 could also regulate other pathological process, such as diabetic cardiomyopathy (DCM). Some researches have been reported that IGF-1 could reduce myocardial damage *via* activating Akt/GSK-3β signaling to inhibit apoptosis and promote metabolizing of glucose [49]. In this regard, targeted delivery of basic fibroblast growth factor (bFGF) also shows significant potential [50]. Like VEGF, the traditional delivery approaches of IGF-1 also face many issues, such as short half-life, low blood concentration, and low targeting. Both of them require new delivery systems to overcome these challenges. Furthermore, growth differentiation factor-15 (GDF15), oncostatin M (OSM), and neuromodulin (NRG) are also efficacious in enhancing myocardial function and may be used as targeted protein drugs for CVD [51–53]. But the above candidates still require new delivery pathways to realize their potential ability to treat CVD.

#### 3.1.2. Small molecule drugs

In the treatment of CVD, widely used of small-molecule drugs are primarily novel inhibitors, most of which need to enter cells and interact with specific proteins, enzymes, or receptors to modulate intracellular signaling pathways, thereby exerting therapeutic effects. However, these small-molecule drugs could not reach a satisfying intracellular absorption rate by conventional drug delivery methodologies, which implies a reduction in target efficiency and bioavailability [54]. The advent of advanced targeted delivery systems, such as nanoparticles and liposomes, has engendered a plethora of strategies for the delivery of small-molecule drugs, enabling them to reach the lesion sites with greater precision and efficiency, thereby enhancing their therapeutic efficacy [4].

Milrinone (MRN), a commonly used clinical drug for heart failure (HF), demonstrates inadequate bioavailability and brief systemic retention time due to its absence of target specificity [55]. However, by utilizing angiotensin II (AT1) peptide-conjugated human serum albumin nanoparticles (AT1-HSA-MRN-NPs) as carriers and modifying their surfaces with specific ligands, MRN can be targeted to the myocardium, allowing it to more effectively exert its positive inotropic and vasodilatory effects for the treatment of HF [55]. During MI, Poly(ADP-ribose) (PAR) can be produced by Poly(ADP-ribose) polymerase-1(PARP-1), leading to accelerated cardiomyocyte apoptosis, energy depletion, inflammatory infiltration, and so on [56]. The PARP-1 inhibitor AZ7379 can reduce the intracellular content of PAR, and improve the prognosis of MI. Compared with free AZ7379, cardiomyocyte-specific (I-1) liposomes-AZ7379 complex demonstrates higher efficiency in inhibiting PARP-1, providing greater advantages in improving the prognosis of MI [57]. Besides targeting cardiomyocytes, monocytes and macrophages are also ideal targets for CVD treatment: studies have also shown that the METTL3 inhibitor STM2457, delivered *via* red blood cell-derived microvesicles, can fundamentally alter the infiltration, differentiation, and functional behavior of monocytes within cardiac tissues, thereby reducing inflammation and fibrosis in the heart [58].

Consequently, a range of small-molecule pharmaceuticals that have been extensively utilized in the management of CVD, encompassing enzyme inhibitors, receptor agonists/antagonists, ion channel modulators, and even chemical inhibitors, such as TAK-242-NP, may also be enhanced in finally their therapeutic efficacy through the implementation of innovative delivery systems [22,59–61].

### 3.2. Types of carriers in targeted for delivery

#### 3.2.1. Exosomes

**Figure 1.**
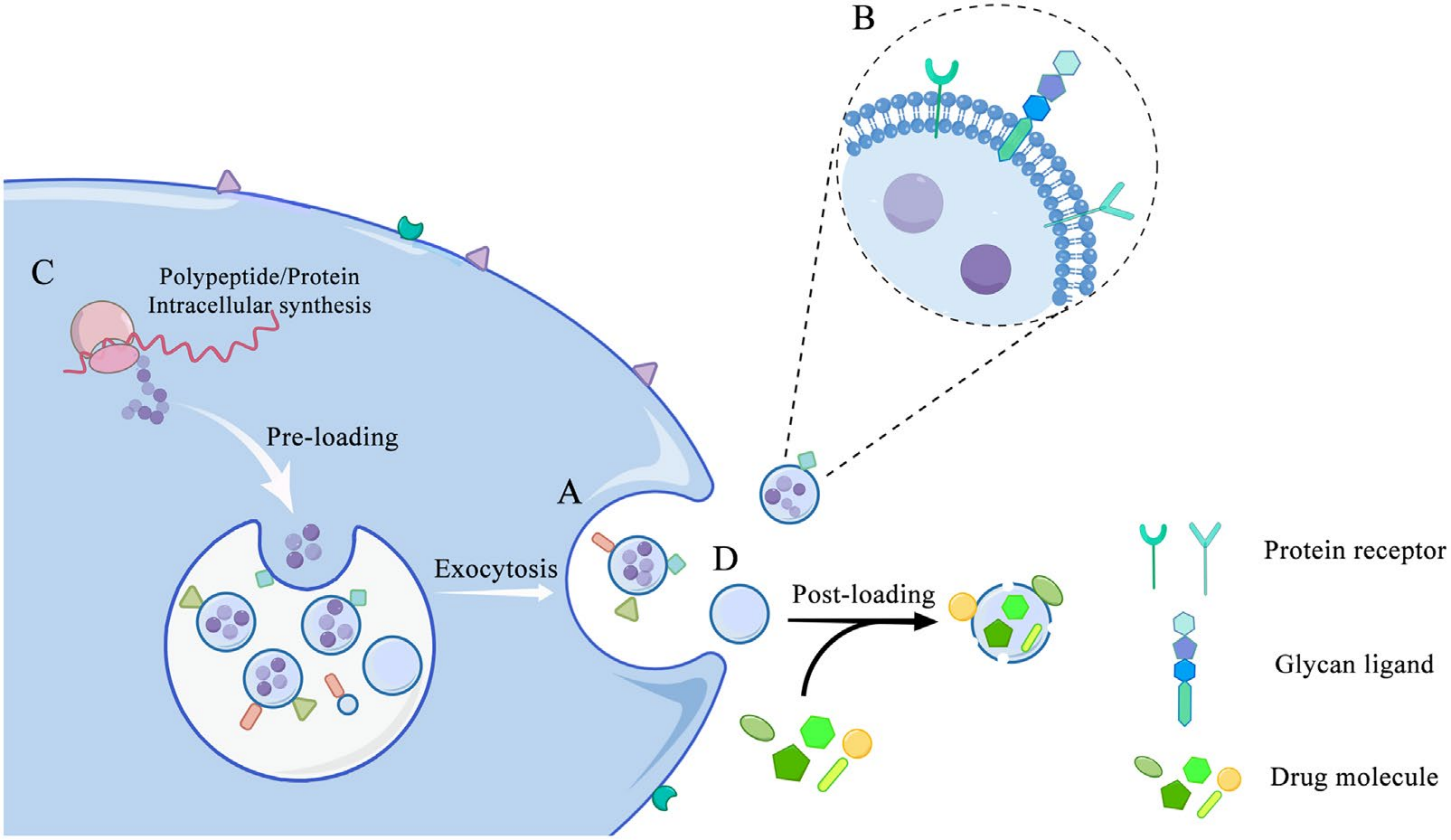
Exosomes secretion, structure, and drug loading process. (A) MVBs release exosomes to the extracellular space by exocytosis. (B) Exosomes display signaling molecules on their surface, such as protein receptors and glycan ligands. (C) In the pre-loading (intracellular loading) technology of exosomes, protein/peptide drugs are synthesized inside the cell, transferred to exosomes, and secreted out of the cell. (D) In the post-loading (extracellular loading) technology of exosomes, the exosomes are collected first and then loaded with drug molecules using physical or chemical means. MVBs: Multivesolar bodies.

Exosomes are bilayer lipid membrane microcapsules secreted by cells, which are usually 30–150 nm in diameter and contain complex biomolecules such as proteins, lipids, and nucleic acids [62,63]. They originate from early endosomes formed by cellular endocytosis, which undergo acidification and maturation into late endosomes by receiving Golgi apparatus and other components, and then develop into multivesolar bodies (MVBs). Finally, MVBs fuse with the cell membrane and release them to the extracellular space (Figure 1A) [64]. Due to the unique origin and structure, exosomes can be ideal targeted carriers. Firstly, exosomes are small, so they can cross various biological barriers, such as the blood-brain barrier [65]. Moreover, compared with artificial drug carriers, self-derived exosomes isolated from dendritic cells have better biocompatibility and low immunogenicity, which brought greater safety in clinical applications [66]. Most importantly, exosomes possess natural intercellular communication functions. Their lipid bilayers can be inserted into various signaling molecules such as proteins and sugars, and these signaling molecules can be directly targeted to specific cells (Figure 1B) [67,68]. Therefore, the use of exosome carriers undoubtedly brings the possibility to improve the accuracy and efficiency of treatment.

However, since the production of exosomes relies on living cells, their separation and purification processes are complex, and it is difficult to load drugs, this limits the application of exosomes to a certain extent [69]. Serum as a nutritional support is crucial in the process of living cells secreting and producing exosomes, but at the same time, serum itself contains exosomes as well as other unknown components that may affect the subsequent isolation and purification of the produced exosomes. Although the application of serum-free media can better solve the above problems, current research also shows that the application of such media will increase the content of reactive oxygen species (ROS) and stress proteins, which also has a negative impact on the quality of exosome production [70]. The low efficiency of exosome production is also a problem that cannot be ignored: less than 1 μg of exosomes can be isolated from 1 mL of culture medium, which is far from the amount of 10-500μg required for mouse experiments [71]. Compared with animal experiments, clinical treatment requires more exosomes, so how to improve the production efficiency of exosomes is an urgent problem to be solved. As the active components secreted by cells, how exosomes load therapeutic drugs is also a key issue. Pre-loading (intracellular loading) technology refers to cargo loading during exosome biosynthesis, where exosomes are loaded in donor cells before being released into the extracellular environment (Figure 1C). The advantage of this method is that it does not destroy the structure of the exosomes and maintains their intact biological activity [69]. However, the drug loading efficiency of pre-loading technology is relatively low, and it is only suitable for drugs that can be synthesized in cells, such as nucleic acids and protein/peptide drugs. In contrast, post-loading (extracellular loading) is a potential option for synthetic drugs. In the post-loading method, the drug loading is preformed after exosome isolation (Figure 1D). Cargoes can be packaged into exosomes or adsorbed to the surface of exosomes by means of ultrasound, electroporation, extrusion and surface treatment. However, such approaches may damage the exosome membrane or cause exosome aggregation, thereby reducing delivery efficiency and introducing the risk of immunogen exposure [69]. Nevertheless, it’s believed that with the progress of technology and the development of clinical trials, the deficiency of exosomes will be gradually overcome, which is expected to become valuable drug delivery carriers.

#### 3.2.2. Liposomes

**Figure 2.**
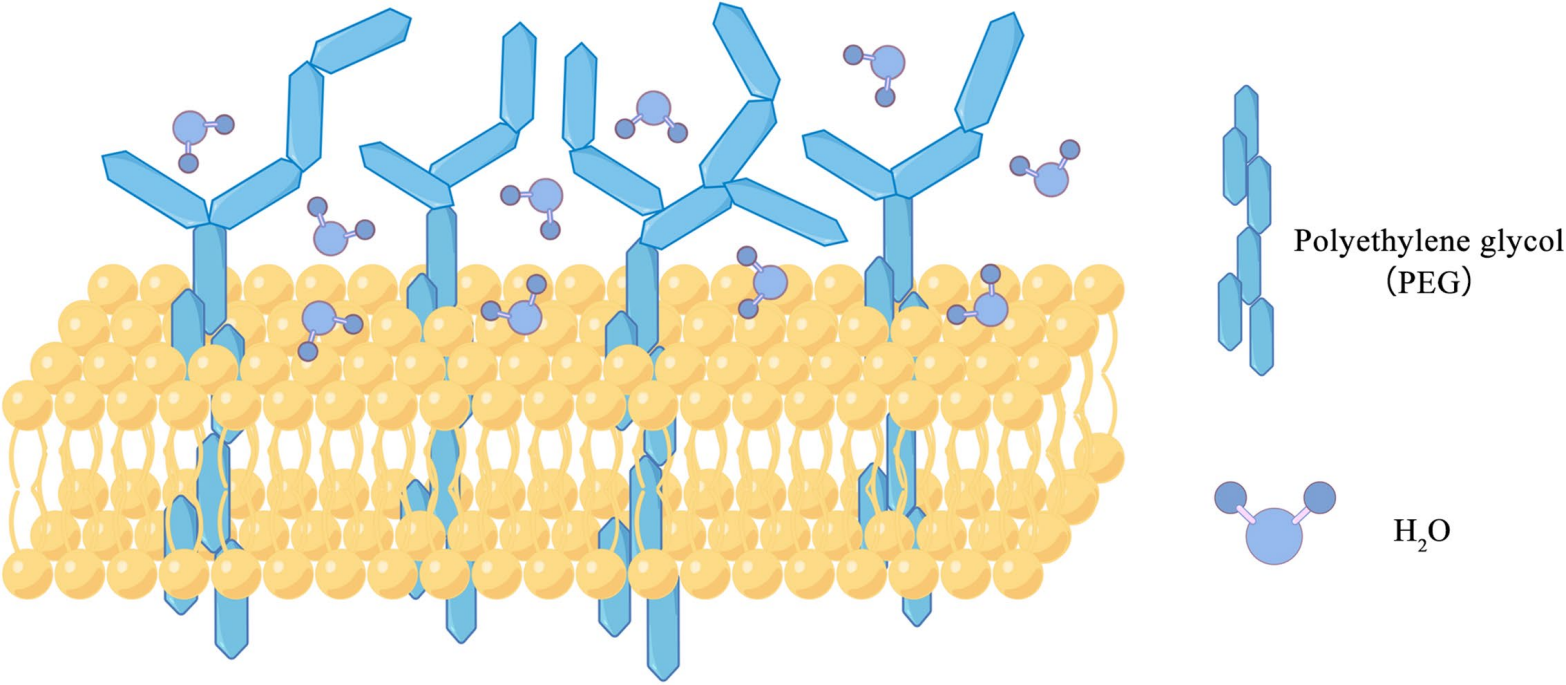
Phospholipid bilayer membrane structure of PEGylated liposomes. PEG molecules are embedded in the liposome bilayer to enhance membrane stability. The PEG branched chains outside the membrane can form a hydrophilic layer after cross-linking, which resists the destruction by plasma proteins and antigen adsorption. PEG: Polyethylene glycol.

Liposomes are artificially synthesized nanoscale vesicles [72]. Similar to exosomes, liposomes also have a bilayer membrane structure, mainly composed of phospholipids and cholesterol, but the difference is that liposomes also contain an aqueous phase core [73]. Due to their unique structure, liposomes are able to load both lipid-soluble drugs (embedded in lipid bilayer) and water-soluble drugs (located in the core of the aqueous phase), effectively preventing drugs dilution, degradation or inactivation in the blood, which can improve the efficiency of drugs delivery. Therefore, liposomes also widely used in the field of drugs delivery [74]. Moreover, liposomes are superior to exosomes in terms of preparation and large-scale production. The preparation of liposomes is relatively easy and can also be mass-produced through methods such as continuous flow technology and microfluidic technology [75–77]. Even more surprisingly, numerous studies indicate that introducing polyethylene glycol (PEG) modifications can significantly enhance the delivery capabilities of liposomes, making them more versatile and adaptable for clinical applications [74]. PEG, enhances liposome stability by forming ‘anchors’ embedded within the phospholipid bilayer and a mesh-like ‘coat’ on the membrane surface. Among them, the enhanced stability primarily stems from the membrane surface ‘coat’ formed by PEG long-chain molecules, which effectively insulates the liposomes from the plasma lipoproteins that can emulsify and destroy them (Figure 2). Concurrently, this PEG layer also reduces the adsorption of liposome drugs by immunoglobulins, delaying phagocytic clearance by the mononuclear phagocyte system (MPS), thus prolonging the half-life of the drug and increasing the blood drug concentration.

In addition, it is worth mentioning that some studies focused on the combination of ultrasound-mediated drug delivery technology and liposome drug delivery also bring surprises to patients with CVD [78,79]. This approach combines the precision of ultrasound with the biocompatibility of liposomes [80,81]. Using the cavitation effect, mechanical effect and thermal effect of ultrasound, and through its specific frequency, intensity or irradiation time, liposomes can release drugs at the target site, so as to achieve the localization of drugs or even the timed and quantitative release. For example, a study on the prevention of DCM demonstrated that the cavitation effect of ultrasound-targeted microbubble destruction (UTMD) technology can cause liposomes carrying fibroblast growth factor (bFGF-lip) to rupture and release bFGF in the hearts of diabetic rats. This approach enabled precise spatiotemporal control of bFGF release [50]. At present, liposome-packaged drugs are developing rapidly in clinical applications. Some drug complexes have entered the clinical trial stage and achieved therapeutic effects superior to those of traditional drugs. The clopidogrel-aspirin drug complex packaged by liposomes demonstrated high sustained release capacity in clinical trials, and was superior to traditional antiplatelet therapy with fewer complications [82]. Similarly, in a clinical study on the therapeutic effectiveness of liposomes-packaged methotrexate, liposomes-methotrexate complex effectively reduced the area of myocardial infarction due to its highly efficient anti-inflammatory ability, lower dosage and side effects [83]. In summary, combining ultrasound-mediated drug release technology with liposomes or other carriers, such as the aforementioned exosomes, provides a new precise, efficient, and low-toxicity method for the treatment of CVD [19,84].

#### 3.2.3. Hydrogels

**Figure 3.**
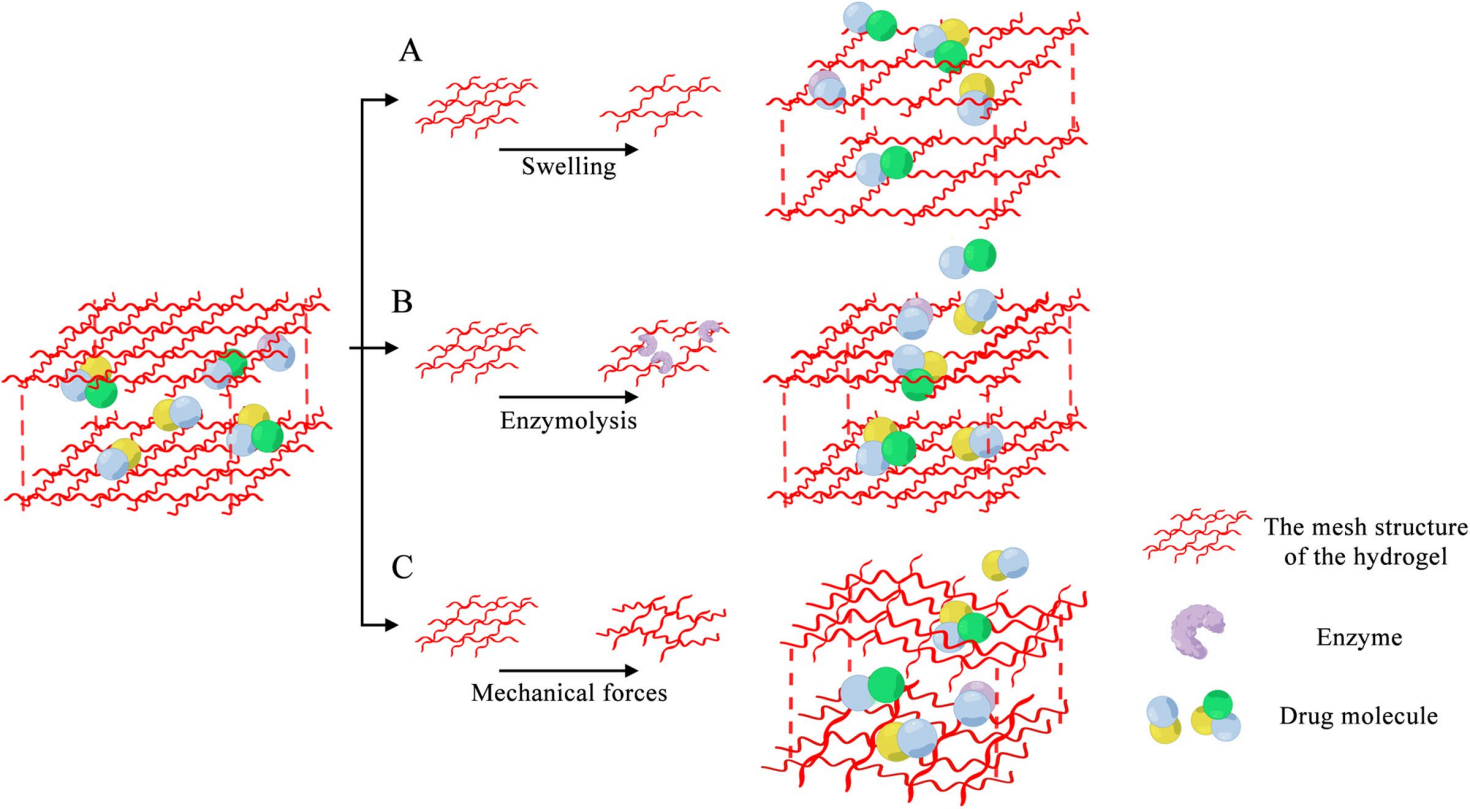
The mechanisms of drugs release from hydrogels. (A) The mesh size of the hydrogel increases upon swelling. (B) Enzymolysis causes hydrogel disintegration, resulting in an increased mesh size. These structural modifications facilitate the diffusion of drug molecules through the enlarged mesh, promoting their release into the target tissue. (C) Mechanical forces deform the hydrogel microstructure, leading to mesh expansion.

Hydrogels are a type of three-dimensional network structure material formed by the cross-linking of hydrophilic polymers through physical or chemical means, and they have the unique property of rapidly swelling upon absorbing water without dissolving themselves [85–87]. The hydrophilic polymers constituting the hydrogels may be derived from either natural or synthetic. Natural polymers, including collagen, HPD, and chitosan offer high biocompatibility and biodegradability [88–91], while synthetic polymers like polyacrylamide, polyvinyl alcohol, and PEG provide structural controllability and superior mechanical properties [92–94]. As an attractive drug delivery carrier, hydrogels, with their unique physical and chemical properties, have created a distinctive drug controlled-release mechanism, which is determined by the proportional relationship between the drug diameter and the size of the hydrogel mesh [95]. When drug molecules are smaller than the hydrogel mesh size, their release into surrounding tissues occurs primarily *via* passive diffusion, though this process is inherently difficult to spatially or temporally control [96]. Conversely, drugs larger than the mesh will be immobilized until factors that cause the mesh size to increase are present, thus controlling drug release [95]. Controlled release through swelling is one of the fundamental mechanisms. When the mesh size of the hydrogel increases due to water absorption and expansion, the drug molecules can be released from the gel by diffusion (Figure 3A) [97,98]. The rate of drug release is affected to some extent by the rate of swelling of the hydrogel, with faster swelling indicating faster release [99]. The dissolution mechanism is also an important pathway for hydrogel drug release. In this mechanism, the hydrogel material itself (the polymer backbone or crosslinks) will gradually degrade *in vivo*, thereby releasing the embedded drugs, and the degradation process is mainly chemical degradation, such as hydrolysis or enzymatic hydrolysis (Figure 3B) [100–103]. Hydrogels can also be subjected to external mechanical forces like pressure and stretch to change the mesh size, thus adjusting the rate and amount of drug release (Figure 3C) [104]. After the mechanical stress, the hydrogel can return to its original state, and the release rate will be changed accordingly [104]. In addition, the introduction of specific functional groups can also confer stimulus responsiveness to hydrogel carriers, enabling them to respond to changes in external environment, such as temperature, pH, and light, providing hope for meeting the diverse therapeutic needs of CVD [105–107].

Despite demonstrating immense application potential due to their numerous outstanding properties, hydrogels present a highly complex preparation process compared to other carriers during practical implementation. The fabrication of hydrogels requires precise, coordinated control of multiple parameters—including temperature, pH, and crosslinking density—which interact closely and influence one another [108–110]. Even minor fluctuations in any single parameter can significantly alter hydrogel properties. At the molecular level, this intricate interplay stems from the synergistic effects of these parameters on the hydrogel network structure. For instance, temperature variations alter the kinetic behavior of molecular chains, thereby affecting crosslink formation efficiency [111]. Meanwhile, pH fluctuations may disrupt hydrogen bonding or ionic interactions between functional groups, further compromising the stability of the crosslinked network [112]. Additionally, optimizing crosslink density requires not only balancing monomer concentration and crosslinker dosage but also tailoring the design to specific preparation methods and application scenarios [113]. This complexity of multi-parameter synergistic regulation imposes significant technical barriers on hydrogel fabrication processes and poses substantial challenges for clinical translation. Consequently, simplifying hydrogel preparation techniques and enhancing process controllability represent critical issues demanding urgent resolution for hydrogel carriers.

#### 3.2.4. Inorganic nanomaterials

Nanomaterials can generally be classified as organic or inorganic. As previously introduced for liposomes, organic nanomaterials are highly biocompatible and readily degradable [114]. These characteristics make them ideal carriers in the field of drug delivery. However, organic nanomaterials have shortcomings in terms of stability, shelf life, and drug encapsulation, which undoubtedly limits their application [115]. Compared with organic nanomaterials, inorganic nanomaterials exhibit higher stability and drug loading capacity, as well as mechanical plasticity, magnetic properties and photosensitivity, making them attractive for drug delivery application [116,117]. Among inorganic nanocarriers, Mesoporous Silica Nanoparticles (MSNs), Gold Nanoparticles (AuNPs) and Magnetic Nanoparticles (MNPs) are widely studied at present. With their own characteristics, they occupy a place in the research field of CVD treatment.

Benefiting from their high specific surface area and tunable pore structure, MSNs exhibit significant advantages in the field of drug delivery. The high specific surface area provides abundant binding sites for drug adsorption, thereby substantially enhancing drug loading capacity [118,119]. Relevant studies have shown that the specific surface area of MSNs can typically reach hundreds of square meters per gram, a characteristic that contributes to their exceptional performance in drug loading [120]. Furthermore, the pore size of MSNs can be flexibly adjusted by precisely controlling synthesis conditions, ranging from approximately 2 to 50 nm [121]. This tunability allows for the selection of an appropriate pore size based on the dimensions of drug molecules, enabling efficient drug encapsulation and storage. For instance, smaller pores can form an effective physical barrier to prevent premature drug leakage, while larger pores provide more ample space for drug molecules, thereby improving drug loading efficiency [122]. Consequently, through scientifically rational optimization of pore design, MSNs not only ensure drug stability in complex environments but also achieve higher drug loading capacity, offering a solid and critical foundation for enhancing the overall performance of drug delivery systems for CVD.

AuNPs also represent a promising cardiovascular drug delivery platform, offering versatile loading strategies including physical adsorption, covalent conjugation, encapsulation [123–125]. Compared with their high specific surface area and biocompatibility, AuNPs are even more remarkable for their excellent photothermal conversion performance. The photothermal conversion performance of AuNPs originates from localized surface plasmon resonance (LSPR): under specific wavelength light (such as near-infrared light) irradiates AuNPs, the free electrons on their surface can resonate and absorb light energy, which is subsequently converted to heat energy through a non-radiative relaxation process [126]. This highly efficient photothermal conversion mechanism has been extensively studied for the treatment of atherosclerosis (AS), namely photothermal therapy [127–129]. More appealing, the performance also enables light-controlled drug release with precise spatiotemporal regulation [130]. A multifunctional nanoparticle (AuNRs-SiO_2_/RSNO/DS, GSNPD) developed by Yuqing Lu research team demonstrates this controlled release potential well [131]. This nanoparticle features a gold nanorod core and a silica shell, with therapeutic agents loaded into the mesopores of the silica *via* either physical adsorption or chemical conjugation. Upon near-infrared light irradiation, the photothermal effect generated by the gold nanorod induces thermal expansion of the silica shell, thereby triggering the release of the encapsulated drugs [131]. Furthermore, by combining AuNPs with liposomes, engineered thermosensitive liposomes can intelligently utilize this photothermal conversion capability. Under NIR irradiation, the plasmonic nanoparticles deposited on the liposome surface produce mild localized hyperthermia, which induces a phase transition in the lipid bilayer of the liposome [36,132]. This enhances membrane permeability and facilitates the release of the encapsulated drug payload.

MNPs are typically composed of iron (Fe), cobalt (Co), nickel (Ni), or their oxides (e.g. Fe_3_O_4_, γ-Fe_2_O_3_), and their core strengths lie in the superparamagnetism and magnetoresponsiveness. Utilizing superparamagnetism, MNPs can absorb electromagnetic waves under an alternating magnetic field to generate heat for hyperthermia. Once the magnetic field is removed, the magnetism of MNPs dissipates, effectively preventing residual *in vivo* toxicity [133]. Moreover, by virtue of their magnetic responsiveness, MNPs can be directed to move towards the target site, such as the ischemic region of the heart, under the guidance of an external magnetic field, achieving precise targeted delivery [134–136]. Such functional characteristics offer a promising magnetic targeting strategy to address the challenges of low cell retention and transplantation efficiency associated with traditional stem cell-based therapies for CVD [137]. For instance, MSCs or cardiac progenitor cells (CPCs) can be labeled with superparamagnetic iron oxide nanoparticles (SPIONs). Under the guidance of an external magnetic field, these labeled cells can be precisely directed to and enriched within the diseased heart tissue, thereby providing a novel approach for regenerative therapy in the target area [138]

Despite the remarkable progress achieved in the research of inorganic nanomaterials, there are still several bottlenecks impeding their clinical translation. First and foremost, the long-term toxicity assessment of inorganic nanomaterials is insufficient, especially the potential accumulation risk of metal ions [139]. Secondly, in terms of large-scale preparation, there are notable discrepancies between batches in laboratory-prepared inorganic nanomaterial carriers, and breakthroughs are yet to be made in achieving consistent shape control and surface modification for industrial-scale production [140]. Moreover, only a handful of relevant studies have progressed to the preclinical stage. Among the FDA-approved nanomedicines, there is even a gap in the indications for CVD. Consequently, to facilitate the transition of inorganic nanomaterial carriers from the laboratory to the clinic, it is imperative to address critical issues such as safety, scale-up production, and clinical validation.

#### 3.2.5. Engineered bacterial carriers

Engineered bacteria have emerged as drug delivery carriers through genetic engineering, nanotechnology, and other means, providing innovative solutions for the delivery of drugs [141,142], especially protein drugs, for CVD treatment. The delivery system based on engineered bacteria shows incomparable advantages in targeting and local sustained drug release compared with traditional drug delivery methods (Table 1). Some bacteria (such as attenuated Salmonella) have active chemotactic ability, which can sense inflammatory or hypoxic signals and actively migrate to atherosclerotic plaques, ischemic myocardium, and other sites. Such a capability overcomes the low efficiency of passive enrichment of conventional nanoparticles [143]. With the help of the engineered bacteria’s protein synthesis system, therapeutic molecules such as IL-10 can also be continuously expressed and released at the target site to achieve long-term sustained release, thereby avoiding repeated drug delivery [144]. Moreover, through the design of engineered bacteria promoters (such as hypoxia-responsive HIF-1α promoter and inflammation-sensitive NF-κB promoter), the timing of engineered bacteria to express protein drugs can be controlled, which is expected to realize the on-demand release of drugs at the site of lesions.

**Table 1.** Comparison of advantages of engineered bacterial carriers and conventional carriers.

	Engineered bacterial carriers	Conventional carriers
Targeted localization mechanism	Active chemotaxis and environmental response	Passive EPR effect or ligand-receptor binding
Duration of medication	Synthesized at the lesion site continuously	Many drugs have short half-lives and require frequent dosing
Microenvironmental regulation ability	Dynamic regulation of immunity/metabolism	Regulating ability was weak (mainly negative feedback)
Cost of production	Low (mature fermentation process)	High (e.g. antibody-drug conjugates)

EPR: Enhanced permeability and retention.

Engineered bacteria can not only be used as a carrier for the production and transportation of protein drugs, but also can cooperate with stem cell transplantation, potentially enabling the directed differentiation of cardiomyocytes for MI treatment. Pseudomonas aeruginosa carries the type III secretion system (T3SS) that can form a syringe-like complex, and then directly secrete effector proteins and inject them into host cells [145]. Fang Bai’s team used the modified attenuated P. aeruginosa to inject transcription factors directly into embryonic stem cells (ESCs) *via* the T3SS system and efficiently induce their differentiation into cardiomyocytes [146]. This work lays the foundation for engineering bacterial carriers to deliver protein substances such as transcription factors without compromising genomic integrity. However, there are still some risks associated with the delivery of therapeutic drugs by bacterial infection of host cells. On the one hand, antigens carried by bacteria themselves may trigger a strong immune response after entering the host, leading to a local or systemic inflammatory storm, which will not only weaken the therapeutic effect, but also may cause damage to normal tissues and organs. On the other hand, even after attenuated treatment, bacteria may still regain virulence or acquire new pathogenic abilities under some unexpected conditions, and then multiply and spread *in vivo* uncontrollably, which would destroy the normal physiological functions of host cells, and even cause unpredictable infectious diseases. Therefore, before such drug delivery strategies are put into clinical application, more comprehensive and in-depth safety assessment and mechanism research must be carried out to effectively avoid risks and ensure treatment efficacy.

#### 3.2.6. Low-molecular-weight protein/peptide and antibody-mediated targeted drug delivery carriers

Engineering drug molecules through modification of low molecular weight proteins or peptides has become a key strategy to break through the bottleneck of traditional drug delivery [147]. By mimicking the natural receptor-ligand interaction mechanism, this technology can achieve efficient drug enrichment in target tissues while reducing systemic side effects. Moreover, the molecular weight of the peptide is usually only thousands of daltons, which can be directly linked to the drug as a ‘molecular label’ and guides drug binding to the target, thereby eliminating the complex packaging steps and simplifying the production process [148,149]. Besides localization, Cell-Penetrating Peptides (CPPs), a class of low molecular weight peptide chains, can cross the cell membrane and directly guide various types of therapeutic agents such as small molecule drugs and proteins into the cell [150–152]. Their application has changed the dilemma that traditional macromolecular drugs are difficult to penetrate the cell membrane, and has significantly broadened the treatment methods. For example, the BH4 peptide, derived from the anti-apoptotic domain of Bcl-2 protein, exhibits limited ability to penetrate cell membranes directly due to its hydrophilic nature and macromolecular characteristics. However, when conjugated with four distinct CPPs—Tat, (RXR)_4_, Bpep, and Pip2b—the BH4 peptide can be efficiently delivered into cardiomyocytes, where it exerts therapeutic effects [153]. Furthermore, CPPs can also be covalently linked to other therapeutic agents to facilitate their direct intracellular delivery to cardiomyocytes, thereby offering additional strategic opportunities for the treatment of CVD [151].

Low molecular weight proteins or peptides have good penetration ability and offer significant advantages in guiding drugs to penetrate the blood-brain barrier [154]. However, due to the short binding fragments to target proteins, their binding ability and accuracy are weak. By contrast, antibody-mediated targeted drug carriers have better specificity and are more stable [155]. Cardiomyocytes may express different characteristic molecular markers in different diseases, which provides the possibility of antibody-mediated targeted drug therapy. The expression level of P-selectin in normal cardiomyocytes is extremely low, but it is increased in myocardium surface after MI, providing an important target for MI targeted therapy [156]. Studies have shown that Anti-P-selectin coated model drug carriers can be heavily enriched in infarcted cells, utilizing the above target [157]. In addition, antibody-mediated targeted markers also play an important role in the early diagnosis of myocardial diseases. EDA^+^ fibronectin (EDA^+^ Fn), as an early marker of chronic rejection after heart transplantation, is hardly expressed in healthy myocardium [158]. Anti-EDA^+^ Fn antibodies can carry markers, such as I-124 and fluorescent dye DY-682, to label chronic rejection myocardial tissues, which is of great significance for the early detection of chronic rejection lesions. Meanwhile, the antibody can also carry drugs to treat the rejected myocardium, suppressing the local immune response and delaying the onset of rejection [158].

Low-molecular-weight proteins/peptides and antibodies can also act as modifying groups, binding to the surfaces of the above-mentioned carriers such as liposomes, exosomes or inorganic nanomaterials, guiding them to transfer towards the target tissue (Table 2). In this way, relevant drugs can be more precisely located to the lesion, reducing the occurrence of side effects.

**Table 2.** The difference between low molecular targeted peptide and antibody in the process of targeted delivery of drugs.

	Low molecular targeted peptides	Antibody
**The ability to bind to targeted substances**		
Targeted binding ability	High	Middle to low
Risk of off-target	Low	High
**Distribution range and dispersal capacity**		
Molecular weight	High (more than 150 kDa)	Low (1 to 5 kDa)
Ability to distribute	Low (difficult to penetrate various barriers)	High (able to penetrate various barriers)
**Pharmacokinetic**		
Half-value period	Long	Short
Organ of metabolism	Liver	kidney
Stability	High	Low
**Safety of medication**		
Risk of immunogenicity	High (especially non-human antibodies)	Low (can be reduced by optimizing sequence)
Bystander immune effector function	None	ADCC; CDC
**Production and cost**		
Mode of production	Complex (dependent living cells)	Simple (direct chemical synthesis)
Cost of production	High	Low
Drug carrying capacity	Middle	Low

ADCC: antibody-dependent cell-mediated cytotoxicity; CDC: complement-dependent cytotoxicity.

## 4. Targeted drug delivery application for major disease

### 4.1. The development of targeted drugs in CVD treatment

In the field of CVD treatment, research on targeted delivery systems designed for protein-based and small-molecule drugs has been increasingly advancing, with many studies now reaching the preclinical stage. These investigations demonstrate significant potential to transform existing treatment paradigms and herald a new era of precise, efficient, and personalized interventions for CVD (Table 3).

**Table 3.** Strategies for the application of targeted drug delivery systems in cardiovascular therapy.

Diseases	Carriers	Drugs	Mechanism of action	Target cell	Research Sources
Myocardial infarction	Exosomes	IMTP-CSTSMLKAC	Inhibition of inflammatory response in injured myocardium	Myocardium	[30]
	Liposomes	Adenosine	Rapid dilation of the coronary arteries	Myocardium	[159]
		L‐arginine and FTY720	L‐arginine stimulating endothelial cells to produce nitric oxide and dilating blood vessels; while FTY720 sustained release exerts anti‐apoptotic effects	Vascular endothelium	[160]
		Lutein	Lutein improved mitochondrial function by facilitating the translocation of NDUFS1 from the cytosol to the mitochondria through competitive binding to MDM2.	Myocardium	[161]
	Hydrogels	VEGF	Hydrogels control the localization and release of VEGF to promote angiogenesis	Vascular endothelium	[157]
	Inorganic nanomaterials	Allicin	Inhibition of ferroptosis in cardiomyocytes	Ischemic myocardium	[162]
	Low-molecular-weight protein/peptid	VEGF	Low-molecular-weight targeted peptides link VEGF to control its localization to promote angiogenesis	Vascular endothelium	[163]
		Lutein	Low molecular weight targeting peptides were ligated to the lutein carriers to control its localization	Ischemic myocardium	[161]
		Puerarin	CHP guided the localization of puerarin-liposome complex to ischemic myocardium in order to inhibit apoptosis and ferroptosis	Ischemic myocardium	[164]
Atherosclerosis	Exosomes	LDLR	Rescue of LDLR expression in the blood vessels	Vascular endothelium	[165]
	Liposomes	Rosuvastatin calcium	Promoting plaque stability and improving vascular endothelial function	Vascular endothelium	[166]
	Hydrogels	Dexamethasone	Local high concentrations of thrombin break down the hydrogel to release the drug	Vascular endothelium	[103]
	Inorganic nanomaterials	Heparin	Directed to the thrombosis through magnetic field guidance	Thrombosis	[167]
Cardiac arrhythmia	Liposomes	Amiodarone	Drugs are enriched to the myocardium by passive targeting	Myocardium	[168]
Diabetic cardiomyopathy	Liposomes	bFGF	The bFGF activated the PI3K/AKT signal pathway, causing the reduction of myocardial cell apoptosis and increase of microvascular density	Diseased myocardium	[50]

IMTP-CSTSMLKAC: Ischemic myocardium-targeting peptide CSTSMLKAC, VEGF: Vascular endothelial growth factor CHP: Cardiac homing peptide, LDLR: Low-density lipoprotein receptor, bFGF: Basic fibroblast growth factor.

Among these, MI has attracted the most extensive attention as an indication, and related targeting strategies are particularly diverse. For instance, exosomes derived from MSCs fused with the ischemic myocardium-targeting peptide CSTSMLKAC (IMTP) to form IMTP-exosomes offer a novel and effective approach for MI therapy. IMTP-exosomes can preferentially target ischemic cardiac tissue and deliver proteins and other therapeutic cargo to damaged myocardium, exerting multiple effects such as anti-inflammatory, anti-apoptotic, and pro-angiogenic activities, thereby enabling more precise repair of post-MI injury and improvement of cardiac function. Adenosine, a highly potent vasodilator of coronary arteries, can effectively protect against myocardial damage caused by MI [169,170]. However, its direct administration may lead to severe side effects such as hypotension and arrhythmias, coupled with an extremely short half-life, which limit its clinical application [171]. To overcome these drawbacks, adenosine encapsulated in PEGylated liposomes forms a complex that enables precise coronary vasodilation and alleviates MI symptoms while minimizing systemic adverse effects [159]. As previously mentioned, although VEGF can ameliorate MI through multiple mechanisms, conventional delivery approaches often cause off-target vascular hyperplasia in non-target tissues (e.g. tumors), restricting its clinical utility [43]. To address this, researchers have developed a temperature-sensitive aliphatic polyester hydrogel conjugated with VEGF. The hydrogel precursor remains liquid at room or low temperature and can be injected accurately into the MI area. Upon exposure to physiological conditions (37 °C), it undergoes a sol-gel transition to form a solid gel, achieving localized fixation. As the hydrogel degrades or releases the drug *via* diffusion, VEGF is sustainably released into the tissue, promoting angiogenesis and protecting cardiomyocytes, thereby significantly improving cardiac function while avoiding systemic exposure [172]. Alternatively, a collagen-binding domain (CBD) can be genetically fused to the N-terminus of VEGF to create a CBD-VEGF recombinant protein. Serving as a ‘molecular tag’, CBD specifically recognizes and binds to type I collagen, which is abundant in the MI region, guiding VEGF enrichment at the ischemic site and resulting in enhanced therapeutic efficacy [173]. Moreover, certain biomimetic silica nanoparticles provide new perspectives for MI treatment [122]. For example, neutrophil membrane-coated biomimetic MSNs, through surface-expressed β_2_ integrins, can specifically recognize and actively bind to ICAM-1 molecules highly expressed on activated endothelial cells, enabling efficient targeted delivery to inflammatory sites [162]. In a rat model of myocardial ischemia-reperfusion injury, encapsulation of allicin (AL) within such biomimetic MSNs led to significant accumulation of AL in the infarcted myocardium, inhibition of ferroptosis in cardiovascular endothelial cells, and consequently superior therapeutic outcomes [162]. These preclinical studies, spanning material design and target selection, systematically advance the innovation and development of targeted therapeutic technologies for MI.

Beyond MI-related therapies, targeted delivery technologies for protein-based and small-molecule drugs also demonstrate broad application prospects in the treatment of AS. A particularly noteworthy example is YN001, a novel formulation comprising rosuvastatin calcium encapsulated in liposomes, which has received clinical trial approvals from both the U.S. FDA and China’s National Medical Products Administration (NMPA) and is currently undergoing global Phase Ia clinical studies [166,174]. Modified with hyaluronic acid-PEG-DSPE (HPD), YN001 actively targets CD44 receptors highly expressed in plaque areas, enabling site-specific drug accumulation. It synergistically inhibits AS progression through multiple mechanisms including lipid-lowering, anti-inflammatory, and antioxidant effects, as well as plaque stabilization and improvement of endothelial function [166]. In response to the complex microenvironment of AS lesions, characterized by abnormal mechanical stress, elevated ROS, and infiltrating inflammatory macrophages, a dual-sensitive hydrogel carrier system with mechanical responsiveness and inflammatory targeting has been developed. This system employs a micelle-composite hydrogel framework that enables shear stress-responsive drug release at sites of vascular stenosis through mechano-induced micelle deformation. Simultaneously, it utilizes hyaluronic acid (HA) to specifically bind CD44 receptors on inflammatory macrophages within plaques, achieving precise delivery of drugs such as aspirin to enhance AS treatment [175]. Meanwhile, immune cell infiltration, as a characteristic of AS plaques, can also serve as a therapeutic target. By inhibiting M1 macrophages and promoting the polarization of M2 macrophages, plaques can be effectively stabilized and the probability of fibrous cap rupture can be reduced. The removal of specific subtypes of T cells or B cells through monoclonal antibodies has also shown a good therapeutic effect on arterial plaques [176]. In addition, macrophages, as the main recruitment targets of inflammatory plaques, can also serve as targeted carriers for drugs. By coupling drugs to the surface of macrophages and during their chemotaxis towards plaques, targeted drug delivery to diseased plaques can be achieved [121]. In the realm of inorganic nanomaterials, a conjugate of SPIONs with dexamethasone phosphate (DEXA), designated SPION-DEXA, has shown remarkable efficacy in a rabbit AS model. Under magnetic guidance, SPION-DEXA efficiently accumulates in abdominal aortic plaques, offering an innovative strategy for localized intervention in AS [138]. Furthermore, familial hypercholesterolemia (FH), a major risk factor for AS, is driven primarily by impaired low-density lipoprotein receptor (LDLR) function, resulting in reduced hepatic uptake of LDL cholesterol (LDL-C), elevated plasma LDL-C levels, and vascular lipid deposition [165,177]. Targeting this mechanism, recent studies have innovatively packaged functional LDLR proteins into exosomes. Leveraging the innate targeting ability of exosomes, such as integrin-mediated binding to vascular endothelial adhesion molecules, this approach achieves hepatocyte-specific LDLR delivery. Animal studies confirm that this strategy significantly reduces plasma LDL-C levels and attenuates plaque burden in LDLR-deficient mice, providing a novel biological targeting pathway for the treatment of FH-related AS [177]. It is worth emphasizing that the primary threat of AS stems from thrombotic complications following rupture of unstable plaques, yet current local interventional strategies for thrombosis remain inadequate. Addressing this critical gap, researchers have developed a thrombin-responsive hydrogel delivery system capable of effectively delivering anticoagulants to prevent, treat, and resolve thrombi [178]. By incorporating thrombin-sensitive substrates (e.g. peptide sequences such as LVPRGS or GGRGD) into the hydrogel cross-linking network, high local concentrations of thrombin during thrombus formation selectively cleave these peptides, leading to gel degradation and subsequent release of anticoagulants such as heparin. The released drugs then bind and inhibit thrombin, creating a feedback loop that moderates further peptide cleavage and drug release [178]. This bioinspired cascade-regulation mechanism enables real-time adaptation of drug release to pathological progression, reduces bleeding risks compared to conventional anticoagulant therapies, and maintains effective drug concentrations at the thrombus site, offering a revolutionary solution for the treatment of thrombotic diseases.

Furthermore, in the field of arrhythmia treatment, several antiarrhythmic drugs are associated with significant side effects and suboptimal bioavailability [179,180]. In light of these limitations, targeted delivery systems have demonstrated remarkable potential in mitigating these drawbacks. Amiodarone, one of the key candidate drugs for arrhythmia management, is known to accumulate in multiple tissues, leading to clinically observed adverse effects such as bradycardia, thyroid dysfunction, and pulmonary fibrosis [181–183]. Notably, its tendency to precipitate upon dilution poses a risk of vascular occlusion following intravenous administration, which may compromise drug delivery efficacy, reduce therapeutic outcomes, and even cause severe local tissue damage [184]. However, in one study, researchers developed a thin-film hydration method to prepare amiodarone-loaded liposomes, offering a promising strategy to address these challenges. Using a rat model of ischemia/reperfusion (I/R) injury, they thoroughly investigated the effects of amiodarone-loaded nanoliposomes on lethal arrhythmias. The results indicated that these nanoliposomes specifically targeted the I/R-affected myocardial tissue, significantly enhancing the antiarrhythmic efficacy of amiodarone while markedly reducing its adverse effects compared to conventional administration [168]. Similarly, encapsulation using liposomes or other nanocarriers has been applied to other antiarrhythmic agents such as lidocaine hydrochloride and propranolol, effectively lowering their risk of side effects and thereby expanding the therapeutic options for arrhythmia management [185,186].

Certainly, the discussions above only cover a subset of representative targeting strategies and cannot fully capture the broad and dynamic landscape of targeted delivery in the treatment of CVD. In reality, the field continues to witness the emergence of numerous innovative delivery technologies. These advances demonstrate significant potential not only in improving targeted therapies for common conditions such as MI, AS, and arrhythmias, but also offer promising new avenues for the management of other CVD-related disorders including cardiomyopathies and valvular heart diseases.

### 4.2. The application of targeted drugs in other diseases

Neurodegenerative diseases seriously affect the quality of life of the elderly and even tend to be younger. Seeking innovative treatment methods has become the current research focus. Due to the complexity of the central nervous system, the treatment of neurodegenerative diseases is still dominated by drug treatment.

At present, the development of Alzheimer’s disease is one of the most rapidly studied among neurodegenerative diseases. Thanks to the emergence of new monoclonal antibody targeted drugs, the accumulation of Aβ amyloid protein aggregates in the brain has been cleared specifically, thereby alleviating symptoms such as dementia and hypomnesia. Currently marketed Alzheimer’s specific drugs include aducanumab, lecanemab, and donanemab [187]. It mainly marks Aβ amyloid protein through antigen-antibody interaction and guides macrophages to clear it, achieving the goal of alleviating the symptoms [188].

Besides Alzheimer’s disease, targeted drug therapy for other neurodegenerative diseases is also advancing steadily. Prasinezumab, a targeted drug for α-synuclein (α-syn) aggregates, has also entered clinical trials and shown good results [189]. Therefore, this type of targeted drug has broad prospects in the treatment of neurodegenerative diseases.

Cancer, as the number one disease threatening human health, has received great attention in the development of its treatment methods [190]. Traditional drug treatment lacks effective means to distinguish tumor cells from normal cells, the accumulation efficiency of anticancer drugs in tumor cells is low and the toxic side effects on normal tissues are high [191]. How to achieve efficient targeted enrichment of anti-cancer drugs has become a key area of current drug research. The role of targeted drug delivery through the outer packaging route as a drug carrier is achieved by liposome encapsulation. Among them, the most mature and widely used one is Doxorubicin entrapped in PEGylated liposomes-Doxil^®^. As an anti-cancer drug, doxorubicin’s strong cytotoxic effects have brought about severe side effects, such as cardiotoxicity, bone marrow suppression and hair loss. The side effects of doxorubicin can be significantly reduced by coating with PEGylated liposomes: PEGylation can mask the surface charge of liposomes reducing its liver uptake and interaction with MPS, as well as enhancing their tumor accumulations [192].

The successful application of the aforementioned drugs has greatly boosted researchers’ confidence in the development of cardiovascular targeted drugs. The development of various flexible applications of cardiovascular-targeted drugs has been proven to be a practically feasible engineering problem.

## 5. Administration routes of targeted drugs

The drug administration routes significantly influence drug efficacy *in vivo*. Appropriate administration routes can make drugs work better while reducing the potential for off-target effects, thus decreasing the side effects of the drugs. Therefore, it is essential to identify a suitable administration approach for targeted drugs. Nowadays, the appropriate routes for administering cardiac targeted drugs encompass intravenous injection, local injection, catheter intervention, and surgical implantation, among others [193]. We shall introduce these commonly used methods of administration, as well as their indications, advantages or disadvantages.

### 5.1. Intravenous injection

Due to the large size and complex structure of targeted drug molecules, oral administration needs to face challenges such as digestive tract destruction, difficult absorption, and first-pass effect [194]. Thus, above targeted drugs are not suitable for absorption by oral administration. As an alternative to the oral mode of administration, intravenous injection is one of the most common modes of administration, and many targeted drugs can be administered intravenously with good absorption and potency. Intravenous injection requires that the structure of the drug complexes should not be too large and can successfully pass through the pulmonary capillary network to the myocardial tissue. Therefore, intravenous injection is the preferred mode of delivery for many of the above drugs, including exosomes, liposomes, inorganic nanoparticles, and low-molecular-weight protein/peptide labeled molecular drugs [195]. However, since intravenous drug complexes are distributed in the blood vessels of the whole body, to achieve the goal of enriching drugs in the diseased myocardium, higher requirements are placed on the ability of the drug complexes to bind to the targeted tissue. In addition, the release and enrichment of drug complexes in targeted tissues can also be achieved by intravenous injection with the help of ultrasound-mediated or magnetic guidance [50,167]. In terms of patient compliance and injury, intravenous injection has less damage, which is more suitable for elderly and frail patients or patients who need long-term repeated administration. For areas with weak medical level, this method of administration also highlights its unique value due to its simple and convenient operation.

### 5.2. Local injection

Some targeted drugs cannot pass through the pulmonary capillary network because of their large structure, so they cannot reach the myocardial tissue after intravenous injection, but deposited in the pulmonary capillary, and even have the risk of embolism. For example, after intravenous injection of MSCs, the cells will deposit in the pulmonary capillary network [196]; temperature-sensitive phase-change hydrogel, which turns into a solid phase after injection into the vivo, is also not suitable for intravenous injection [172]. The advantages of local injection, such as intramyocardial or pericardial injection, are well developed when faced above drug complexes. What′s more, local injection is easier to achieve lesion area drug delivery and the local drug concentration enrichment. For drug complexes that are difficult to label, such as nanoparticles loaded with stem cells, intramuscular injection can achieve precise colonization in the lesion area. Whereas temperature-sensitive phase change hydrogel is suitable for pericardial injection. In this way of drug delivery, the hydrogel after phase transition can not only achieve targeted drug delivery and controlled drug release, but also act as an isolation layer to block the necrotic area from contacting the pericardium, thus reducing the occurrence of adverse prognostic events such as fibrin adhesion [197]. Despite local injection has many advantages, it also associated with greater risks than intravenous injection. Intramyocardial injection may cause bleeding and even pericardial tamponade due to necrotic myocardium was be penetrated, and may also rise the incidence of ventricular aneurysm in the long term; while pericardial injection requires strict control of the dosage, too high dosage is more likely to cause cardiac tamponade in the weak heart. Therefore, local injection of target drug puts forward higher requirements for the operator’s ability, and the indications should be strictly controlled.

### 5.3. Catheter intervention and surgical implantation

As the gold standard for the treatment of MI in the acute phase, catheter intervention can accurately relieve vascular obstruction [198]. While catheter intervention is carried out to recanalize the blood vessels, the administration of relevant targeted drugs can enable the drugs to rapidly accumulate in the obstructed myocardium. This not only enables the recanalization of the obstructed site but also saves the ischemic myocardium and promotes vascular growth. Therefore, when performing catheter intervention to recanalize blood vessels, using catheters to deliver targeted drugs to the obstructed site can better leverage the advantages of both.

For hydrogel drugs (such as hydrogel mesh, etc.), due to their large volume, the general administration methods cannot reach the lesion site, so surgical implantation has become the only way of drug delivery. However, the high risk of surgical implantation, especially for patients with serious CVD such as MI, limits the application of this kind of drugs to a certain extent. With the development and popularization of minimally invasive surgery, surgical implantation may also become a promising adjuvant treatment option.

### 5.4. Other administration routes

In addition to the above routes of administration, there are rarer but non-invasive methods of administration, such as inhalation administration [199]. Through the modification of transmembrane peptides, the drug can be guided to pass through the pulmonary capillary into the bloodstream and reach the heart to exert its effect [200]. This route is completely non-invasive and suitable for early emergency treatment and repeated drug administration, but there are still some problems, such as low drug absorption rate, and further research is needed to overcome the shortcomings.

### 5.5. Different administration routes are suitable for different diseases and drugs

For different cardiovascular diseases, hemodynamic changes can affect the efficiency of drug administration. For myocardial infarction patients, because of the insufficient blood perfusion in the infarction area, drugs are difficult to reach the infarction site through systemic administration. Therefore, in the acute stage of myocardial infarction, the local administration route is more suitable to be combined with interventional surgery. In contrast, for thrombosis patients, due to the variable location, wide distribution and small size of the thrombi, local administration has disadvantages such as narrow coverage. At this point, the systemic administration route through intravenous injection highlights its advantage in widely locating thrombus sites. Therefore, choosing the appropriate administration method is a key step in treatment (Table 4).

**Table 4.** The compatibility among diseases, carriers, drugs and administration routes.

Diseases	Carries	Target tissue	Administration routes	Targeted approach
Myocardial infarction	Exosomes Liposomes Inorganic nanomaterials	Infarcted myocardium	Intravenous injection /Local injection /Catheter intervention	Active targeting and Magnetic field positioning
	Low-molecular-weight protein/peptid	Infarcted myocardium	Intravenous injection	Passive targeting
	Hydrogels	Infarcted myocardium	Surgical Implantation	Stimulation -responsive targeting
Atherosclerosis	Exosomes Liposomes Inorganic nanomaterials	Vascular endothelium and plaques	Intravenous/Local injection	Active targeting
	Hydrogels	Thrombosis	Local injection	Stimulation -responsive targeting
Cardiac arrhythmia	Liposomes	Myocardium	Intravenous	Active targeting
Diabetic cardiomyopathy	Liposomes	Diseased myocardium	Intravenous/Local injection	Active targeting

The above administration routes have their own application scope and advantages. According to the patient’s condition and the type of targeted drugs, the appropriate administration method can be selected to maximize the efficacy of the drug and reduce the side effects.

## 6. Challenges and future perspectives

At present, targeted drug therapy is a popular research direction and development of new drug research. Many highly effective drugs have been developed one after another, and there are also many new achievements in targeted drugs for the treatment of CVD. However, there are still many challenges in bringing targeted drugs into clinical use on a large scale, and there is still room for improvement to overcome the limitations.

### 6.1. Challenges faced by targeted drugs

The preparation of targeted drugs is related to the safety of clinical application. The production of exosomes mainly relies on the *in vitro* culture of cell. This process also faces problems such as low production efficiency and obvious batch effects. How to achieve controllable quality industrial production is the key for exosome-based targeted drugs to enter clinical application. The industrial production of bioactive drugs such as insulin provides a good reference for exosomes. In this process, transforming the existing production system and expanding the production scale might be the key to improving production efficiency and reducing batch effects. In addition to the above-mentioned problems such as production efficiency, complex process and batch effect, the compatibility between the targeted drug and the carrier is also a factor that cannot be ignored before the targeted drug is to enter the clinical application. For example, some lipophilic small molecule drugs may be inserted into the liposome membrane as an emulsifier, emulsifying and destroying the bilayer membrane structure, making the targeted drug structure destroyed and ineffective, and reducing the potency of the drug [201]. Besides, chemical reactions may occur between the drug and the carrier, resulting in structural changes of the drug and thereby affecting its efficacy. For example, some drugs contain active groups such as amino groups and carboxyl groups, and reactions such as oxidation, hydrolysis, and esterification may occur when they are in contact with some oxidizing or acid-basic carrier materials [202]. Therefore, the compatibility of drug and carrier is also a key consideration in the design and development of drug delivery system, which directly affects the stability of drug and the final therapeutic effect and safety.

The prerequisite for drugs to exert their therapeutic effect is that it can avoid being cleared by the immune system. As the targeted drug complex is a large exogenous foreign body, it is easy to adsorb substances such as opsonin during the distribution in the circulatory system and be phagocytosed by macrophages [203]. At present, there are mainly three approaches to reducing the clearance of drugs by the immune system: 1, Adding PEG chains to reduce the possibility of opsonin adsorption [27]; 2, By introducing markers to achieve the transmission of ‘self-identification’ signals, such as CD47, the signal of ‘Don’t eat me’ is sent to macrophages [204]; 3, Directly encapsulate nanoparticles with cell membranes extracted from one’s own cells such as red blood cells and white blood cells. This ‘bionic camouflage’ makes the nanoparticles look like a part of the body, almost perfectly deceiving the immune system and greatly prolonging their circulation time in the blood [205].

Targeting accuracy is also a key factor affecting the clinical application of targeted drugs. An ideal targeted drug delivery system would release the drug in an appropriate rate at a specific time point and target site to maximize efficacy and minimize side effects. However, due to the complexity and unpredictability of the *in vivo* environment, it is still difficult to achieve the precise drug release in time and space [206,207]. Hydrogel can also undergo drug release depending on pH value or temperature, but this is affected by the degree of lesion or disease process in the focal area [208]. For example, after MI, the pH value of the lesion area at different time points is different, and the control of drug release by hydrogel requires personalized drug customization to meet the needs of different patients or different period [209]. This also brings challenges to the design and production of targeted drugs. In addition, the accuracy of external magnetic field or ultrasound-mediated drug localization technology still needs to be improved, and the side effects caused by leaked drugs need to be further evaluated.

In the meantime, the long-term toxicity of targeted drugs still needs to be further detailed assessment. Further studies are needed to verify whether the immunogenicity of macromolecular complexes such as liposomes, exosomes, and hydrogels can lead to allergy after long-term use; inorganic nanomaterials carriers with a diameter of less than 5.5 nm can be excreted directly by glomerular filtration; the biliary system can excrete particles with larger diameters (10–12 nm), but particles with a larger diameter cannot be excreted by any route and will accumulate in the body for a long time [210,211]. In view of the situation, further studies are needed to clarify the cumulative toxic and side effects of inorganic nanomaterials *in vivo*. The potential risk of long-term application of engineered bacterial carriers is that the auxotrophic strains break through the nutrient limitation and gain uncontrolled proliferation through ‘reversion mutation’ or ‘compensatory mutation’, resulting in serious infection [212,213]. Therefore, the long-term toxicity evaluation of targeted drug carriers should be cautious.

The transportation and storage conditions of targeted drugs are also key issues affecting their application. Since targeted drugs are usually biologically active complexes, high requirements are put forward for their transportation and storage conditions. Whether a targeted drug can maintain a high activity before use is related to the therapeutic effect of the drug. Full-temperature cold chain transportation or on-site deployment and application are expected to solve related problems, but they also bring problems such as cost and quality control. However, in the modern medical system that has popularized full cold chain transportation, this problem seems to be easily solved. At present, with the establishment and maturation of the vaccine transportation and storage system, various vaccines that need to be refrigerated have been widely adopted in developing countries. Targeted drugs that require cold chain transportation can be stored and transported through this system without any additional investment.

### 6.2. The development prospect of targeted drugs

In order to meet the above challenges, targeted drug delivery systems can be optimized by combining a variety of emerging technologies. The current direction of medical development is to emphasize prevention and early intervention of diseases. CRISPR-Cas9 system provides precise gene editing ability. Combined with targeted drug delivery technology, it can achieve genetic modification of specific tissues and reduce the risk of disease from the level of genetic susceptibility. For example, for people with coronary LDL receptor deficiency, targeted delivery of CRISPR-Cas9 system to the diseased vessel to repair the defective gene in the local tissue can not only achieve disease prevention, but also reduce the off-target effects of gene editing on the whole body. To achieve the above goals, more accurate receptor-ligand recognitions are needed to enable precise positioning of targeted drugs. Under this background, artificial intelligence (AI) -assisted targeted ligand design is gradually emerging: biological big data analysis and deep learning can deal with the complex structure of cell surface receptors, making it possible to design highly specific ligands. In this way, researchers can design targeting ligands more efficiently, greatly improve the efficiency of targeted drug delivery, and optimize the design parameters of targeted delivery systems. In summary, combined with cutting-edge technologies such as gene editing and AI, targeted drug delivery systems are expected to further improve the therapeutic effect and safety in the future, and open up broader prospects for the treatment of CVDs.

## 7. Conclusions

The core value of targeted drug delivery system lies in its ability to precisely regulate drug distribution and release, which makes it show great potential in various diseases. This review clearly showed the components and key points of this system: the appropriate therapeutic drugs are combined with delivery carriers, and the appropriate administration methods form a systematic engineering, and each link has a crucial impact on the final efficacy. In terms of therapeutic drugs, proteins and small molecule drugs can rapidly intervene in the metabolic state of cells, and can quickly play a role in critical heart diseases to rescue damaged cardiomyocytes. For delivery carriers, many have been widely studied, and even entered the clinical application stage. However, in the face of such a complex variety of carriers, how to select the right carrier to match the required drug synthesis is the key. In addition, according to the state of the patient and the type of carrier, the appropriate administration method can be selected to maximize the effect of the drug.

The clinical transformation of targeted drug delivery systems in CVD is still in early stages, but, the successful application of targeted drug delivery systems in the fields of cancer and neurodegenerative diseases has also provided many valuable experiences. Its systematic design concept and precise regulation ability have laid a theoretical foundation and technical path for treatment breakthroughs in this field. In the future, by deepening the understanding of disease heterogeneity, optimizing the drug-carrier synergy system, and promoting the transformation of more preclinical research into clinical trials, targeted delivery systems are expected to provide more efficient and personalized treatment solutions for a variety of complex diseases.

## Data Availability

Data sharing is not applicable to this article as no new data has been generated and update in this study.
